# Diagnostic Value of Metagenomic Next-Generation Sequencing for Pneumonia in Immunocompromised Patients

**DOI:** 10.1155/2022/5884568

**Published:** 2022-12-01

**Authors:** Jun Li, Chao-E. Zhou, Shan-Chen Wei, Li-Na Wang, Ming-Wei Shi, Chun-Ping Sun, Lian-Jun Lin, Xin-Min Liu

**Affiliations:** Department of Geriatrics, Peking University First Hospital, Peking University, Beijing 100034, China

## Abstract

**Introduction:**

The diagnosis of pulmonary infection and the identification of pathogens are still clinical challenges in immunocompromised patients. Metagenomic next-generation sequencing (mNGS) has emerged as a promising infection diagnostic technique. However, its diagnostic value in immunocompromised patients needs further exploration.

**Purposes:**

This study was to evaluate the diagnostic value of mNGS compared with comprehensive conventional pathogen tests (CTs) in the etiology of pneumonia in immunocompromised patients and immunocompetent patients.

**Methods:**

We retrospectively reviewed 53 patients who were diagnosed with pneumonia from May 2019 to June 2021. There were 32 immunocompromised patients and 21 immunocompetent patients with pneumonia who received both mNGS and CTs. The diagnostic performance was compared between mNGS and CTs in immunocompromised patients, using the composite diagnosis as the reference standard. And, the diagnostic value of mNGS for mixed infections was further analyzed.

**Results:**

Compared to immunocompetent patients, the most commonly pathogens, followed by *Cytomegalovirus*, *Pneumocystis jirovecii* and *Klebsiella pneumoniae* in immunocompromised patients. Furthermore, more mixed infections were diagnosed, and bacterial-fungal-virus coinfection was the most frequent combination (43.8%). mNGS can detect more types of pathogenic microorganisms than CTs in both groups (78.1% vs. 62.5%, *P* = 0.016and 57.1% vs. 42.9%, *P* = 0.048). The overall diagnostic positive rate of mNGS for pathogens was higher in immunocompromised patients (*P* = 0.002). In immunocompromised patients, a comparable diagnostic accuracy of mNGS and CTs was found for bacterial, fungal, and viral infections and coinfection. mNGS had a much higher sensitivity for bacterial infections (92.9% vs. 50%, *P* < 0.001) and coinfections (68.8% vs. 48.3%, *P* < 0.05), and it had no significant advantage in the detection of fungal infections, mainly due to the high sensitivity for *Pneumocystis jirovecii* in both groups.

**Conclusion:**

mNGS is more valuable in immunocompromised patients and exhibits apparent advantages in detecting bacterial and mixed infections. It may be an alternative or complementary diagnostic method for the diagnosis of complicated infections in immunocompromised patients.

## 1. Introduction

Pneumonia is a global public health problem due to its high morbidity and mortality [1, 2]. Pneumonia in immunocompromised hosts can lead to more susceptibility to different pathogens ranging from common to opportunistic pathogens. The causative agent of up to 60% of infectious diseases remains unknown based on clinical phenotype [3, 4]. Finding the causative pathogens in the general population is associated with decreased morbidity and mortality. Comprehensive conventional pathogen tests (CTs) (blood, sputum, bronchoalveolar lavage fluid (BALF), etc.), including bacterial, fungal, acid-fast bacilli smears, cultures, and antigen/antibody-based testing were routinely used in clinic. However, the current diagnostic efficiency of traditional pathogen detection is only 30%–40% [5]. It was limited by time consumption, repeated inspection, and poor detection rate.

Metagenomic next-generation sequencing (mNGS) is a high-throughput sequencing method that can widely analyze the microbiome of clinical samples and has the advantages of direct specimen detection, broad pathogen coverage, and rapid feedback [6, 7]. It has been reported to identify pathogens in various specimen types such as bloodstream respiratory, central nervous system, and focal tissue infection [8–10]. Moreover, a large number of rare pathogens have been detected by the mNGS method, which provides a timely and effective way for the diagnosis of intractable cases [11–13]. However, the research on the diagnostic performance of mNGS in immunocompromised pneumonia is still relatively limited [14–17]. The majority of studies have focused on the comparison of the diagnostic performance of mNGS and blood culture etiology, and few studies have investigated the difference between CTs and mNGS. There is also a lack of studies comparing the etiological diagnostic performance of mNGS in immunocompromised and immunocompetent patients.

This study was to evaluate the diagnostic value of mNGS compared with CTs in the etiology of pneumonia in immunocompromised patients and immunocompetent patients. We aimed to analysis the diagnostic performance of mNGS in bacterial, fungal, and virus infections. Furthermore, the diagnostic value of mNGS for mixed infections was further analyzed.

## 2. Materials and Methods

### 2.1. Study Design and Participants

**Figure 1:**
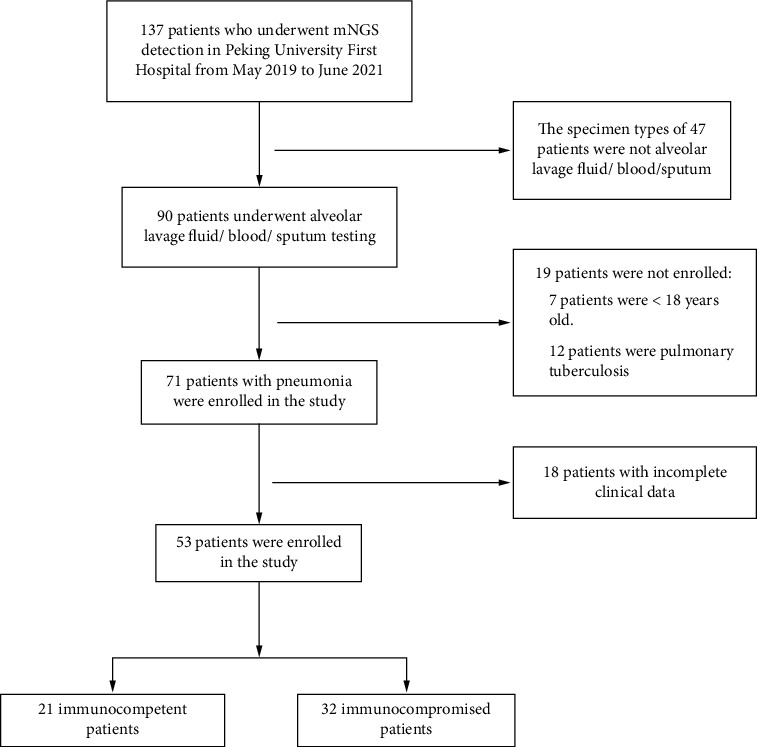
Flow diagram of the study. A total of 137 patients performed mNGS detection. 53 patients were screened after excluding 84 patients who did not meet the inclusion criteria. mNGS: metagenomic next-generation sequencing. There were 21 immunocompetent patients and 32 immunocompromised patients.

**Figure 2:**
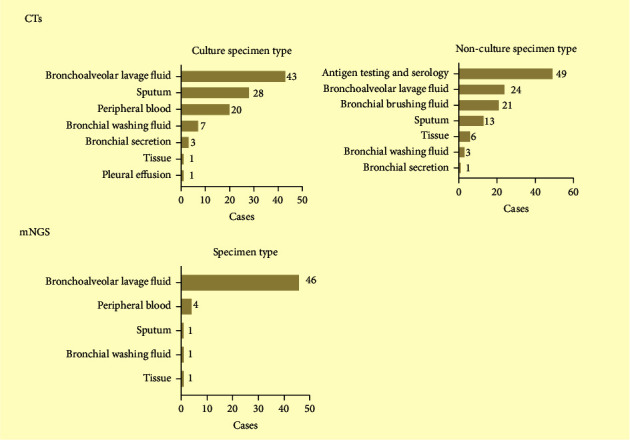
Distribution of sample types in CTs and mNGS. CTs were divided into culture and nonculture group. In the culture group, a total of 103 samples were collected from 43 bronchoalveolar lavage fluid, 28 sputum, 20 peripheral blood, 7 bronchial washing fluids, 3 bronchial secretions, 1 tissue, and 1 pleural effusion. In the nonculture group, a total of 117 samples were included: 49 antigen testing and serology, 24 bronchoalveolar lavage fluid, 21 bronchial brushing fluid, 13 sputum, 6 tissue, 3 bronchial washing fluid, and 1 bronchial secretion. A mNGS test was collected from 46 bronchoalveolar lavage fluid, 4 peripheral blood, 1 sputum, 1 bronchial washing fluid, and 1 tissue. CTs: comprehensive conventional pathogen tests.

A retrospective study recruited 53 study subjects between May 2019 and June 2021 who were immunocompetent and non-HIV immunocompromised adult patients (age ≥ 18 years) with pneumonia (Figure 1). The patients were hospitalized in Peking University First Hospital who were admitted if (1) they had one or more immunocompromised status; (2) they were diagnosed with pneumonia based on the Infectious Diseases Society of America (IDSA)/American Thoracic Society (ATS) criteria [18]; (3) they had BALF and other relevant samples (blood, sputum, tissue, etc.) available for standard procedures (Figure 2); (4) CTs include two or more pathogenic tests (bacterial, fungal, acid-fast bacilli smear and culture, Grocott's methenamine staining (GMS), (1,3)-*β*-D-glucans (*G* test), galactomannan antigen (GM), TB-spot, and GeneXpert, etc.) (Figure 2). Immunocompromised status should meet one or more of the following [19]: (1) using corticosteroid therapy (≥20 mg/d prednisone or equivalent, continuous ≥14 days or cumulative dosage of 600 mg), disease-modifying antirheumatic drugs, biological immune modulators, or other immunosuppressive agents for more than 30 days; (2) active malignancy or receiving cancer chemotherapy; (3) solid organ transplantation; (4) hematopoietic stem cell transplantation; (5) primary or acquired immune deficiency diseases. Patients were excluded if they met any of the following criteria: (1) age < 18 years old; (2) HIV infection; (3) clinical data were incomplete. Baseline data were collected from the clinical electronic medical record system, including demographic characteristics, hospital length of stay, comorbidities, immunosuppressive states, clinical symptoms, signs, laboratory indicators (blood routine, procalcitonin, hypersensitive C-reactive protein, erythrocyte sedimentation rate, T lymphocyte subsets), microbiological testing, chest imaging, treatment process, and prognosis.

Patients were divided into an immunocompromised group and an immunocompetent group according to immune function. Moreover, the diagnostic value of mNGS and CTs was compared (including two or more conventional testing methods).

### 2.2. Microbiological Testing and Pathogenic Analysis

The specimen (BALF, blood, sputum, bronchoalveolar lavage fluid, and tissue) was divided into small portions and paired for CTs and mNGS testing (Table S1). Some specimens were sent to CapitalBio Corporation, Beijing, China, for the mNGS analysis. Once the laboratory receives the specimens, the testing company will process the specimens immediately, performing nucleic acid extraction, library preparation, high-throughput sequencing, and bioinformatics analysis. The interpretation of results mainly depends on relevant standard procedures. A positive result from mNGS data would be if the coverage rate of bacteria (*Mycobacteria* excluded) or viruses (species level) scored 10-fold higher than that of any other microorganism, and the coverage rate of fungi was 5 times higher than that of any other fungus [4, 20]. For *Mycobacterium tuberculosis* (MTB), it was considered positive when at least 1 read was mapped to the species or genus level [21]. The other specimens were sent to our hospital's microbiological laboratory using CTs for pathogenic analysis.

### 2.3. Conventional Pathogenic Tests

In addition to the etiological testing of the above-mentioned specimens, other related samples (sputum, blood and urine) for CTs, including bacterial, fungal smear and culture, acid-fast stain, GM, *G* test, *Cryptococcus neoformans* antigen, TB-spot, GeneXpert, real-time polymerase chain reaction (PCR) (*Cytomegalovirus* (CMV), *EB virus*, *influenza virus*, *respiratory syncytial virus*, *Legionella*, *Mycoplasma*, and *Chlamydia spp*), GMS (*Pneumocystis jirovecii*), and direct microscopic examination of specimens. The results of the CTs were interpreted according to the standard procedure [22, 23].

### 2.4. mNGS Testing

#### 2.4.1. Sample Collection, Processing, and Nucleic Acid Extraction

Specimens were collected and immediately sent to the testing company (CapitalBio Corporation, Beijing, China) for about 3 hours. Total nucleic acid was extracted from 3-4 ml of BALF, sputum, or pleural effusion and from 5 ml of blood samples. In brief, blood samples were collected in tubes of 5 ml of ethylenediaminetetraacetic acid and plasma was separated at 1600 g for 10 min. 3-4 ml of sputum, bronchoalveolar lavage solution (BALF), or pleural effusion was collected in sterile tubes. Sputum samples were liquefied using 0.1% dithiothreitol for 30 min at room temperature.

#### 2.4.2. Library Preparation and High-Throughput Sequencing

DNA was extracted using the QIAamp DNA Microbiome Kit (Cat#51704, Qiagen, Germany). RNA was extracted using the QIAamp Viral RNA Mini Kit (Cat#52904, Qiagen, Germany). Extracted RNA was reverse transcribed using random primers, and cDNA was combined with DNA from the same clinical sample for sequencing library preparation. The pooled nucleic acids were digested to a size of 200–300 bp, and a sequencing library was constructed by end repair, adapter ligation, and PCR amplification. Sequencing templates were further prepared using the One Touch2 System (Life Technologies, CA, USA) and sequenced on a BioelectronSeq 4000 sequencer (CapitalBio Corporation, Beijing, China) after quality control.

#### 2.4.3. Bioinformatics Analysis

Firstly, quality control was taken from the raw sequencing data and reads less than 50 bp in length and of low quality were removed. The remaining sequencing data were depleted of human host sequences by mapping to the human reference genome grch38 using Bowtie2 software (https://bowtie-bio.sourceforge.net/). Then, the nonhuman sequences contained 13992 bacterial, 1659 fungal, 13000 viral, and 287 parasitic pathogens, which were classified by simultaneously aligning genomic sequence databases downloaded from the National Center for Biotechnology Information (https://ftp.ncbi.nlm.nih.gov/genomes/) and Pathosystems Resource Integration Center (PATRIC) databases (Bacterial and Viral Bioinformatics Resource Center | BV-BRC). Following the above steps, we reviewed data from various types of samples from healthy individuals and calculated relevant reference values (including hit reads number and coverage of all bacteria, fungi, viruses, and parasites detected) to identify suspected pathogens. Moreover, the pathogens detected in the water samples of the negative control were removed from the results of the clinical samples. Lastly, the list of suspected pathogens, the number of reads, and genome-level coverage are counted as final etiological test results.

#### 2.4.4. Final Clinical Diagnoses

The final clinical diagnosis was based on clinical manifestation, laboratory tests, chest radiology, microbiological tests (including CTs and mNGS), and antibiotic treatment response. Pathogens were classified into 4 categories according to mNGS: (1) definite: BALF or blood or sputum mNGS result is consistent with results from CTs (BALF/blood/sputum culture, nucleic acid-based testing, and pathological examination) performed within 7 days of specimen collection, based on clinical manifestation, chest radiology, and laboratory findings; (2) probable: mNGS pathogen is likely the cause of pneumonia according to clinical, radiologic, or laboratory findings, but the mNGS result [10, 24] was consistent with CTs; (3) possible: mNGS result has pathogenic potential and is consistent with clinical presentation but other explanation is more likely; (4) unlikely: pathogens detected by mNGS has pathogenic potential but is not consistent with clinical presentation [24]. The clinical pathogenic microorganisms were defined as (1) + (2).

#### 2.4.5. Statistical Analysis

Continuous variable data, if normally distributed, were expressed using the mean (mean ± standard deviation (SD)). We used the median (interquartile range) if the data were not normally distributed and compared the two groups using a *t*-test or Mann–Whitney *U* Tests. Categorical variables were expressed as percentages (%), and comparisons between two groups were made by Fisher's or chi-squared test. All data analyses above were performed using SPSS 23.0. Then, we used the clinical composite diagnosis as the gold standard. Sensitivity, specificity, accuracy, positive predictive value (PPV), negative predictive value (NPV), and 95% confidence intervals were calculated using VassarStats and GraphPad software. The McNemar test was used to compare the diagnostic performance of CTs and mNGS.

## 3. Results

### 3.1. Demographic Characteristics

Based on the inclusion criteria, we excluded 84 patients. Fifty-three patients with pneumonia were screened out of 137 patients. Twenty-one immunocompetent patients and 32 immunocompromised patients were included in the study. The patient characteristics are presented in Table 1. There were no significant differences between immunocompetent and immunocompromised patients in terms of age (60 vs. 62.5, *P* = 0.339), gender (13 vs. 19, *P* = 0.854), smoking (6 vs. 14, *P* = 0.265), drinking (5 vs. 13, *P* = 0.206), oxygenation index (355.7 vs. 291.6, *P* = 0.821), and the incidence of mechanical ventilation (2 vs. 9, *P* = 0.167). The hospital length of stay was longer in immunocompromised patients than in immunocompetent patients (26.5 vs. 8, *P* = 0.043). Among serological indicators, lymphocyte count (0.5 vs. 1.1, *P* = 0.044), serum creatinine (89.8 vs. 64, *P* = 0.007), and B cell count (33.1 vs. 107, *P* = 0.029) were lower in immunocompromised patients. 

Among the 53 cases, mNGS and CTs were used to detect the pathogens. Specimens tested by CTs were divided into cultures and noncultures. Among the culture specimens, BALF cultures were performed in 43, sputum cultures in 28, and blood cultures in 20. The other specimens were included by bronchial washing fluid, bronchial secretion, tissue, and so on. In nonculture specimens, antigen testing and serology testing were performed in 49, BALF tests in 24, and bronchial brushing fluid tests in 21. The main samples tested by mNGS are 46 BALF specimens, 4 blood specimens, and 1 sputum specimen (Figure 2).

### 3.2. Pneumonia Pathogens in the Immunocompromised Patients

**Figure 3:**
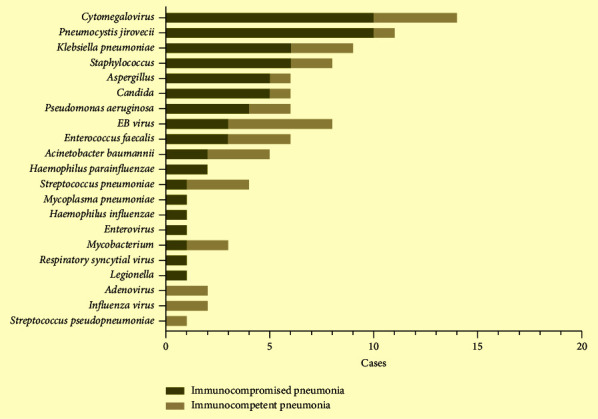
The distribution of pathogens in immunocompromised patients and immunocompetent patients with pneumonia.

**Figure 4:**
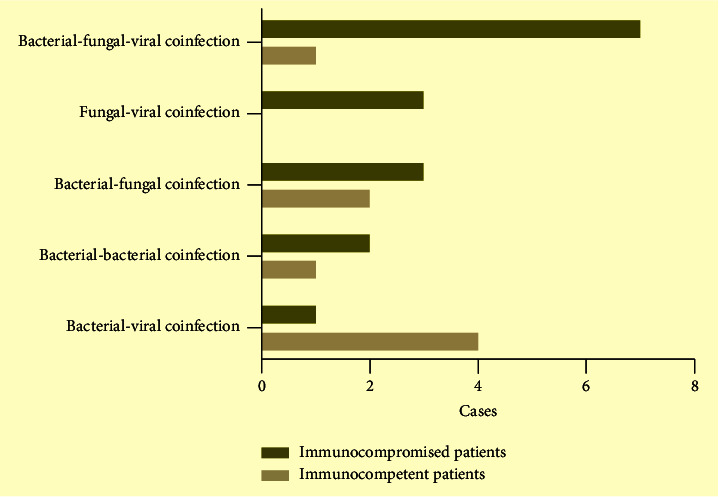
Common combinations of mixed infections in immunocompromised and immunocompetent patients with pneumonia.

Pathogens were detected in 45 of the 53 patients. All detected bacterial, fungal, and viruses are listed in Figure 3. In immunocompromised patients, the most commonly detected bacteria were *Klebsiella pneumoniae*, *Staphylococcus*, and *Pseudomonas aeruginosa*. The most detected fungi were *Pneumocystis jirovecii*, *Aspergillus*, and *Candida* species. And, *Cytomegalovirus*, EB virus, respiratory syncytial virus, and enterovirus were the most frequently detected viruses. In immunocompetent patients, the most commonly detected bacteria were *Klebsiella pneumoniae*, *Streptococcus pneumonia,* and *Acinetobacter baumannii*. The most detected fungus was *Candida*. And the EB virus was the most frequently detected virus. Overall, *Cytomegalovirus*, *Pneumocystis jirovecii*, *Klebsiella pneumoniae*, and *Staphylococcus* were the most detected pathogens in immunocompromised patients. Compared with immunocompetent patients, *Pneumocystis jirovecii* was found more in immunocompromised patients (*χ*^2^ = 3.918, *P*=0.048). Twenty-four (45.3%) patients had mixed infections among 53 patients with pneumonia. Although the comparison of mixed infections between the two groups was not statistically significant, bacterial-fungal-viral infections (43.8%) were the most frequent combinations in immunocompromised patients (Figure 4). A total of 16 patients had mixed infections that occurred in immunocompromised patients, which included 7 bacterial-fungal-viral infections, 3 fungal-viral infections, 3 bacterial-fungal infections, 2 bacterial-bacterial infections, and 1 bacterial-viral infection (Figure S1).

### 3.3. Diagnostic Performance of mNGS in Immunocompromised Patients

**Figure 5:**
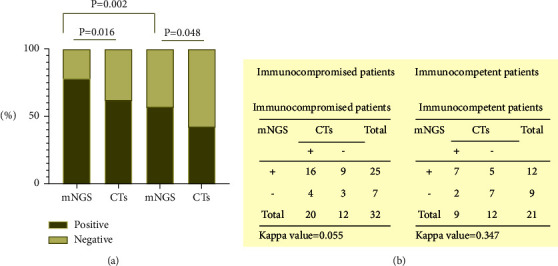
The positivity rate comparison and concordance analysis between mNGS and CTs. (a) The comparisons of the positive rates for mNGS and CTs in immunocompromised patients and immunocompetent patients (*P* < 0.05). (b) Concordance tests and kappa values were shown between mNGS and CTs for immunocompetent patients and immunocompromised patients.

The diagnostic positive rates of mNGS and CTs in the two groups of patients are shown in Figure 5(a). There were statistically significant differences in the positive rates of mNGS and CTs between the two groups of patients (*P* < 0.05). The diagnostic positive rate of mNGS was higher than that in the immunocompetent group (78.1% vs. 57.1%, *P* < 0.05). The kappa values were low in immunocompromised and immunocompetent patients (0.055 and 0.347) (Figure 5(b)).

In immunocompromised patients, mNGS and CTs had comparable diagnostic accuracy rates for different pathogens. mNGS can detect more pathogen species than CTs (Figure S2). It showed a higher sensitivity than CTs (92.9% vs. 50%, *P* < 0.001) in bacterial detection. The PPV and NPV of the mNGS were 72.2% (95% CI, 46.4–89.3%) and 92.9% (95% CI, 64.2–99.6%), respectively. In fungi detection, the sensitivity of mNGS was 100% (95% CI, 51.7–100%) and the specificity of mNGS was 92.3% (95% CI, 73.4–98.7%). There was no significant difference between mNGS with CTs. The PPV and NPV of the mNGS were 75% (95% CI, 35.6–95.5%) and 100% (95% CI, 82.8–100%), respectively, for fungi detection. The sensitivity of mNGS in detecting viruses was 60% (95% CI, 27.4 to 86.3%) and the specificity was 86.4% (95% CI%, 64.0 to 96.4%). The PPV and NPV of the mNGS were 66.7% (95% CI, 30.9–91.0%) and 82.6% (95% CI, 60.5–94.3%), respectively (Table 2).

More mixed pathogens were found in immunocompromised patients. A comparable diagnostic accuracy of mNGS and CTs was found for coinfections. The sensitivity of mNGS was higher than of CTs in detecting the mixed pathogens (68.8% vs. 43.8%, *P* < 0.001). The specificity of mNGS was 87.5% (95% CI, 60.4–97.8%). The PPV and NPV of the mNGS were 84.6% (95% CI, 53.7–97.3%) and 73.7% 82.6% (95% CI, 60.5–94.3%), respectively.

## 4. Discussion

This study described the distribution of pathogens in immunocompromised and immunocompetent patients and compared the diagnostic value of mNGS with CTs in the etiology of pneumonia. Moreover, the effectiveness of different pathogenic diagnoses was explored in immunocompromised patients based on mNGS. We have shown that mNGS is more valuable for pathogenic diagnosis, especially in the detection of bacterial and mixed infections in immunocompromised patients.

Although several studies have reported that the distribution of pathogens and the diagnostic performance of mNGS in different patients [15, 17, 25], the comparative study of mNGS and CTs in immunocompromised and immunocompetent patients with different pathogens is lacking and still controversial. Miao et al. showed that mNGS was not superior to routine microbiological methods for detecting bacteria but had better performance than conventional testing for detecting fungi [21]. However, Fang et al. indicated that mNGS was superior to conventional testing in detecting bacteria and viruses but had no advantages in detecting fungal infections [26]. Peng et al. found that the diagnostic performance of comprehensive conventional tests was similar to that of mNGS for all types of pathogens [17]. Lin et al. found mNGS improved the microbial detection rate of pathogens, compared with comprehensive conventional tests [27]. Unfortunately, previous studies did not identify the definition of comprehensive conventional tests and analyse the diagnostic performance of mNGS versus comprehensive conventional tests in immunocompetent and immunocompromised patients. We selected patients who must have had at least 2 or more traditional pathogenic detection results and compared mNGS with CTs in two groups. The sensitivity, specificity, PPV, and NPV of mNGS and CTs were compared, and the advantages and disadvantages of mNGS methods in the detection of various pathogens were found.

Consistent with previous studies, the distribution of pathogens was different in immunocompetent and immunocompromised patients. In immunocompromised patients, *Cytomegalovirus*, *Pneumocystis jirovecii*, *Klebsiella pneumonia,* and *Staphylococcus* were the most detected pathogens. Compared with immunocompetent patients, more mixed infections were found and bacterial-fungal-viral infections were the most frequent combinations in immunocompromised patients [25]. This may be related to the low immunity of patients, who are more likely to be infected by common and opportunistic pathogens. The study showed that the mNGS method had a higher positive rate than CTs in two groups, which is consistent with previous studies [21, 28]. Furthermore, mNGS had obvious advantages in pathogen detection in immunocompromised than immunocompromised patients (78.1% vs. 57.1%). The kappa values were low (0.055 and 0.347) in two groups, which indicated a lack of consistency. It was due to the diversity of pathogens detected by mNGS.

The strength of our study is that we tried to evaluate the diagnostic performance of mNGS with regard to different types of pathogens and coinfections. We concluded that mNGS had a comparable accuracy rate to that of CTs for the diagnosis of bacterial, viral, and fungal infections and coinfections. However, in immunocompromised patients, mNGS had higher sensitivity than CTs in the detection of bacterial and mixed infections. It could detect many pathogenic bacteria that cannot be detected by CTs. So, mNGS may be used as a routine diagnostic tool for bacterial and mixed infections in immunocompromised patients, although certain disadvantages were pointed out, such as the possibility of the false positive due to the test being too sensitive. Thus, mNGS had no obvious advantages over fungal and viral infections in our study, which was consistent with previous studies [6, 17]. More patients were diagnosed with *Pneumocystis jirovecii* in immunocompromised patients in our study. Jiang et al. reached the conclusion that mNGS had a sensitivity of 100% in diagnosing *Pneumocystis jirovecii* pneumonia (PJP), which was remarkably higher than GMS (25.0%) and the serum *G* test (67.4%) [15]. However, we noticed that the patients had a high sensitivity of CTs (100%) (included G test and GMS) which was similar with mNGS in PJP. Actually, a large sample study is still needed for further exploration. In addition, we explored the diagnostic performance of mNGS in immunocompromised patients with coinfections. mNGS had obvious diagnostic advantages in mixed infections, compared with CTs. We detected mixed infections of bacterial-fungal-viral (*Cytomegalovirus* and *Pneumocystis jirovecii* often coexist with other bacteria), which was the most common pathogen combination.

There are some limitations in the current study. Firstly, the study was a retrospective study with small samples. A further prospective study should be carried out to explore the diagnostic performance of mNGS with different types of pathogens. Secondly, we included various types of immunocompromised patients, and there may be a relationship between the differences in their etiological composition and the diagnostic performance of mNGS. Lastly, we did not assess the guiding value of mNGS in the management of immunocompromised versus immunocompetent patients.

## 5. Conclusion

Overall, the distribution of pathogens was different between immunocompetent and immunocompromised patients. mNGS is a promising alternative or complementary diagnostic method for detecting bacterial and coinfections, especially in immunocompromised patients. It may be an alternative or complementary diagnostic method for the diagnosis of complicated infections in immunocompromised patients.

## Figures and Tables

**Table 1:** Patient characteristics and baseline of two groups.

Characteristics	All patients (*n* = 53)	Immunocompetent patients (*n* = 21)	Immunocompromised patients (*n* = 32)	*P* value
Age (yr)	61 (48.5, 69.5)	60 (43.5, 68.5)	62.5 (54.8, 70)	0.339
Male, *n* (%)	32 (60.4%)	13 (61.9%)	19 (59.4%)	0.854
Hospital length of stay	17 (7, 38)	8 (6, 33.5)	26.5 (12.3, 42.3)	0.043^*∗*^
Tobacco use	20 (37.7%)	6 (28.6%)	14 (43.8%)	0.265
Alcohol abuse	18 (34.0%)	5 (23.8%)	13 (40.6%)	0.206
Oxygenation Index	337.4 (173.9, 427.7)	355.7 (176.5, 430.4)	291.6 (165.0, 434.9)	0.821
Mechanical ventilation, *n* (%)	14 (26.4%)	2 (9.5%)	9 (28.1%)	0.167
Lac (mmol/l)	1.4 (0.9, 2)	1.4 (1.0, 2.1)	1.3 (0.9, 2.2)	0.657
PCT (ng/ml)	0.2 (0.1, 1)	0.2 (0.1, 1.3)	0.3 (0.1, 1.0)	0.796
ESR (mm/h)	52 (23.5, 72.5)	41 (18, 62.3)	53 (26, 78)	0.343
HsCRP (mg/l)	42.3 (8.5, 96.5)	27.6 (2.5, 105.5)	45.7 (16.6, 96.5)	0.227
WBC (109/L)	6.9 (4.4, 9.2)	5.8 (4.4, 8.9)	7.2 (4.25, 9.7)	0.530
Hb (g/L)	102 (87.5, 124.5)	114 (94.5, 129)	100.5 (84.5, 115.8)	0.096
LY (109/L)	0.6 (0.4, 1.25)	1.1 (0.5, 1.4)	0.5 (0.3, 1.05)	0.044^*∗*^
PLT (109/L)	185 (92, 250)	191 (145, 230)	151 (83.5, 265.3)	0.592
Scr (mmol/L)	72.3 (60.3, 165.7)	64 (55.1, 74.7)	89.8 (68.6, 207.8)	0.007^*∗*^
ALT (IU/L)	19.5 (13, 51)	20.5 (11.3, 64)	18.5 (13.3, 44.5)	0.955
AST (IU/L)	22 (18, 49.5)	24.5 (18, 76.8)	22 (18.3, 40.5)	0.658
Alb (g/L)	31.2 (27.2, 36.1)	31.9 (26.6, 39.2)	31.2 (27.5, 35.5)	0.721
LDH (IU/L)	292 (178.5, 476)	192 (151, 431.5)	314.5 (200.3, 496.8)	0.081
CD3+ Tcell count (/*μ*l)	416.4 (256, 845.5)	545 (303, 813.7)	396.8 (182.3, 960.6)	0.549
CD4+T cell count (/*μ*l)	225.2 (105, 534)	302.9 (171.7, 458.6)	164.4 (104.9, 596.1)	0.621
CD8+ T cell count (/*μ*l)	178.2 (109.6, 331.5)	205.2 (124.5, 325.9)	175.3 (79.6, 331.5)	0.658
CD16 + 56 + cell count (/*μ*l)	68.7 (20.9, 146.7)	83.8 (28.1, 154.6)	68.5 (18.4, 146.7)	0.696
B cell count (/*μ*l)	77.9 (13.6, 135.9)	107.1 (75.9, 402.5)	33.1 (11.3, 118.4)	0.029^*∗*^

Abbreviations: PCT, procalcitonin; ESR, erythrocyte sedimentation rate; HsCRP, hypersensitivity-C reaction protein; Lac, lactic acid; WBC, white blood cell; Hb, hemoglobin; LY, lymphocyte count; PLT, platelet count; sCr, Serum creatinine; ALT, alanine aminotransferase; AST, aspartate aminotransferase; Alb, albumin; LDH, lactate dehydrogenase. ^*∗*^*P* < 0.05 was considered statistically significant.

**Table 2:** Diagnostic performance of mNGS and CTs in immunocompromised patients.

	mNGS					CTs				
	Sensitivity (95% CI)	Specificity (95% CI)	PPV (95% CI)	NPV (95% CI)	Accuracy (95% CI)	Sensitivity (95% CI)	Specificity (95% CI)	PPV (95% CI)	NPV (95% CI)	Accuracy (95% CI)
Bacterial pneumonia	92.9^*∗*^ (64.2–99.6)	72.2 (46.4–89.3)	72.2 (46.4–89.3)	92.9 (64.2–99.6)	81.3 (64.3–91.5)	50 (24.0–76.0)	94.4 (70.6–99.7)	87.5 (46.7–99.3)	70.8 (48.8–86.6)	75 (57.7–87.0)
Fungal pneumonia	100 (51.7–100)	92.3 (73.4–98.7)	75 (35.6–95.5)	100 (82.8–100)	93.8 (78.8–99.3)	100 (51.7–100)	96.2 (78.4–99.8)	85.7 (42.0–99.2)	100 (83.4–100)	96.9 (82.9–100)
*Pneumocystis jirovecii*	100 (31.0–100)	100 (85.4–100)	100 (31.0–100)	100 (85.4–100)	100 (87.3–100)	100 (31.0–100)	100 (85.4–100)	100 (31.0–100)	100 (85.4–100)	100 (87.3–100)
Viral pneumonia	60 (27.4–86.3)	86.4 (64.0–96.4)	66.7 (30.9–91.0)	82.6 (60.5–94.3)	78.1 (60.0–90.7)	70 (35.4–91.9)	95.5 (75.1–99.8)	87.5 (46.7–99.3)	87.5 (66.5–96.7)	87.5 (71.3–95.6)
Coinfection	68.8^*∗*^ (41.5–87.9)	87.5 (60.4–97.8)	84.6 (53.7–97.3)	73.7 (48.6–89.9)	78.1 (61.0–89.3)	43.8 (20.8–69.4)	87.5 (60.4–97.8)	77.8 (40.2–96.1)	60.9 (38.8–79.5)	65.6 (48.2–79.7)

Abbreviations: CTs, comprehensive conventional pathogen tests; mNGS, metagenomic next-generation sequencing; PPV, positive predictive value; NPV, negative predictive value; CI, confidence interval. ^*∗*^*P* < 0.05, the difference in the parameter was significant between mNGS and CTs.

## Data Availability

Data will be made available on request to the corresponding author.

## Supplementary Materials

Supplementary Materials
*Figure S1*. Percentage of patients with coinfections with various pathogens in immunocompromised patients. *Figure S2.* The comparison of mNGS and CTs for the detection of different pathogens in immunocompromised patients. *Table S1*. Infection identified by CTs and mNGS.
