# Supplementary material for: Diagnostic Value of Metagenomic Next-Generation Sequencing for Pneumonia in Immunocompromised Patients
Source: Can J Infect Dis Med Microbiol. 2022 Dec 1;2022:5884568. doi: 10.1155/2022/5884568 (PMC9731749; doi:10.1155/2022/5884568)
Supplement: Supplementary Materials — Figure S1. Percentage of patients with coinfections with various pathogens in immunocompromised patients. Figure S2. The comparison of mNGS and CTs for the detection of different pathogens in immunocompromised patients. Table S1. Infection identified by CTs and mNGS. [file 5884568.f1.zip › Supplementary Tables (1).pdf]

Table S1. Infection identified by CTs and mNGS

Patient ID	Sex	Age, year	Underlying disease associated with immunosuppression	Pathogens identified by CTs								Pathogens identified by mNGS
				Sample types	Smear	Culture	Antigen testing and serology	G-test	GM-test	Ultrathin cell smear	Pathology microscopy	mNGS
PT1	Male	41	None	Peripheral blood		√	√	√	√			
				BALF		√						√
PT2	Male	60	Lung cancer	Peripheral blood			√	√	√			
				Bronchial washing fluid	√							
				BALF								√
PT3	Male	67	Acute myelogenous leukemia	Peripheral blood			√	√	√			
				Sputum		√						
				BALF		√						√
PT4	Male	63	Post-renal transplantation	Peripheral blood			√					
				Bronchial washing fluid	√							
				BALF		√				√		√
PT5	Female	37	Cervical cancer	Peripheral blood			√	√	√			
				Sputum		√						
				BALF		√			√	√		√

**PT6**	Female	48	Chronic renal disease	Peripheral blood		√	√	√	√			
				BALF		√				√		√
**PT7**	Male	57	None	Peripheral blood		√	√	√	√			
				Sputum		√						
				Bronchial secretion		√						
				BALF	√	√				√		√
**PT8**	Female	37	Cervical cancer	Peripheral blood			√	√	√			
				Sputum		√						
				BALF		√						√
**PT9**	Female	35	Systemic lupus erythematosus	Peripheral blood		√	√	√	√			
				Sputum	√	√						
				BALF		√			√	√		√
**PT10**	Female	26	Systemic lupus erythematosus	Peripheral blood		√	√	√	√			
				Sputum	√	√						
				BALF		√			√	√		√
**PT11**	Male	61	Monoclonal gammopathy of undetermined significance	Peripheral blood		√	√	√	√			
				Sputum		√						
				Bronchial washing fluid	√							
				BALF	√	√			√	√		√
**PT12**	Male	62	Lung cancer	Peripheral blood			√	√	√			

				Sputum	√	√						
				BALF		√						√
**PT13**	Male	60	Carcinoma of urinary bladder	Peripheral blood				√	√			
				Bronchial washing fluid	√					√		
				BALF	√	√			√			√
**PT14**	Male	65	Multiple myeloma	Peripheral blood		√	√	√	√			
				Sputum		√						
				BALF		√						√
**PT15**	Male	29	Post-renal transplantation	Peripheral blood			√	√	√			
				BALF		√						√
**PT16**	Female	73	Rheumatoid arthritis	Peripheral blood		√	√	√	√			
				Sputum		√						
				BALF		√				√		√
**PT17**	Male	77	None	Peripheral blood		√	√	√	√			
				Sputum		√						
				BALF		√			√	√		√
**PT18**	Male	49	Post-allogeneic hematopoietic stem cell transplantation	Peripheral blood		√	√	√	√			
				Sputum	√	√						
				Bronchial washing fluid	√							
				BALF		√						√
**PT19**	Male	18	None	Peripheral blood			√	√	√			

				BALF		√						√
**PT20**	Male	69	None	Peripheral blood			√	√	√			
				Bronchial secretion		√						
				Bronchial washing fluid		√			√			
				BALF	√	√			√			√
**PT21**	Female	74	Membranous nephropathy	Peripheral blood			√	√	√			
				Sputum	√	√						
				BALF	√	√			√	√		√
**PT22**	Male	62	None	Peripheral blood				√	√			
				Sputum	√							
				Bronchial washing fluid						√		
				BALF		√						√
				Lung tissue							√	
**PT23**	Female	57	ANCA-associated vasculitis	Peripheral blood			√	√	√			
				BALF						√		√
**PT24**	Female	74	Membranous nephropathy	Peripheral blood		√	√				√	√
**PT25**	Female	46	None	Peripheral blood			√	√	√		√	
				BALF		√			√		√	√
**PT26**	Male	70	Classical Hodgkin lymphoma	Peripheral blood			√	√	√		√	
				Bronchial washing fluid	√							
				BALF		√						√

**PT27**	Male	59	ANCA-associated vasculitis	Peripheral blood		√	√	√	√			
				Sputum	√	√						
				BALF	√	√						√
**PT28**	Male	60	None	Peripheral blood			√	√	√		√	
				Bronchial washing fluid	√	√			√			
				BALF								√
				Lung tissue							√	
**PT29**	Male	68	None	Peripheral blood			√	√	√	√		
				Sputum	√	√						
				Bronchial washing fluid	√						√	
				BALF		√			√		√	√
				Lung tissue							√	
**PT30**	Male	70	Lung cancer	Peripheral blood			√	√	√			
				Sputum		√					√	√
**PT31**	Female	57	ANCA-associated vasculitis	Peripheral blood			√	√	√			
				BALF		√						√
**PT32**	Male	82	None	Peripheral blood		√	√	√	√			√
				Sputum		√						
**PT33**	Male	68	Carcinoma of penis	Peripheral blood		√	√	√	√			
				Sputum	√	√						
				Bronchial washing fluid	√						√	
				BALF		√						√
**PT34**	Female	90	Pemphigus	Peripheral blood		√	√	√	√		√	√

**PT35**	Female	49	None	Peripheral blood			√	√	√			
				Bronchial washing fluid	√	√						√
				BALF		√						
				Lung tissue							√	
**PT36**	Female	51	None	Peripheral blood			√	√	√	√		
				Sputum	√	√						
				Bronchial washing fluid	√	√				√	√	
				BALF		√					√	√
**PT37**	Female	37	None	Peripheral blood			√			√	√	
				Sputum	√							
				Bronchial washing fluid	√					√	√	
				Bronchial secretion					√			
				BALF		√						√
**PT38**	Female	46	None	Peripheral blood		√	√					
				Bronchial washing fluid	√							
				Bronchial secretion		√						
				BALF		√					√	√
				Lung tissue		√					√	
**PT39**	Female	80	Interstitial pneumonia with autoimmune features	Peripheral blood				√	√	√		
				Sputum		√						
				BALF		√			√		√	√
**PT40**	Male	63	Myelodysplastic syndrome	Peripheral blood			√	√	√		√	

				Sputum		√					√	
				Bronchial washing fluid	√	√				√		
				BALF							√	√
**PT41**	Male	67	Post-allogeneic hematopoietic stem cell transplantation	Peripheral blood			√	√	√			
				Bronchial washing fluid	√					√		
				BALF	√	√			√			√
**PT42**	Male	28	None	Peripheral blood		√	√	√	√			√
				Sputum		√						
				Bronchial washing fluid	√						√	
				BALF		√						
**PT43**	Female	75	None	Peripheral blood		√	√	√	√			
				Sputum		√						
				Bronchial secretion								
				BALF		√						√
**PT44**	Female	60	ANCA-associated vasculitis	Peripheral blood			√	√	√			
				Sputum		√						
				BALF		√						√
**PT45**	Male	76	Lung cancer	Peripheral blood		√	√	√	√			
				Sputum		√						
				BALF	√	√			√			√
**PT46**	Male	54	Nephrotic syndrome	Peripheral blood			√	√	√	√		
				Sputum	√	√						

				Pleural effusion		√						
				Bronchial washing fluid	√				√			
				BALF		√			√	√		√
**PT47**	Female	61	None	Peripheral blood			√	√	√	√		
				Sputum	√							
				Bronchial washing fluid	√	√				√		
				BALF		√						√
**PT48**	Male	68	None	Peripheral blood				√	√	√		
				Bronchial washing fluid	√					√		
				BALF		√						√
**PT49**	Male	70	Gastric adenocarcinoma	Peripheral blood			√					
				BALF		√			√			√
**PT50**	Male	66	Pemphigus	Peripheral blood			√	√	√	√		
				Sputum		√						
				Bronchial washing fluid	√	√				√		
				BALF								√
**PT51**	Male	31	None	Peripheral blood			√					
				Bronchial washing fluid	√					√		
				BALF		√						√
**PT52**	Female	71	None	Peripheral blood			√	√	√	√		
				BALF	√	√				√		√
**PT53**	Male	60	None	Peripheral blood		√	√	√	√			
				Sputum		√					√	
				Lung tissue							√	√

Abbreviation: CTs: Comprehensive conventional pathogen tests; mNGS:metagenomic next-generation sequencing; BALF: Bronchoalveolar lavage fluid; "√" : It means that the examination was carried out.
