# Supplementary figures and images for: Diagnostic Value of Metagenomic Next-Generation Sequencing for Pneumonia in Immunocompromised Patients
Source: Can J Infect Dis Med Microbiol. 2022 Dec 1;2022:5884568. doi: 10.1155/2022/5884568 (PMC9731749; doi:10.1155/2022/5884568)

**Figure S1.**

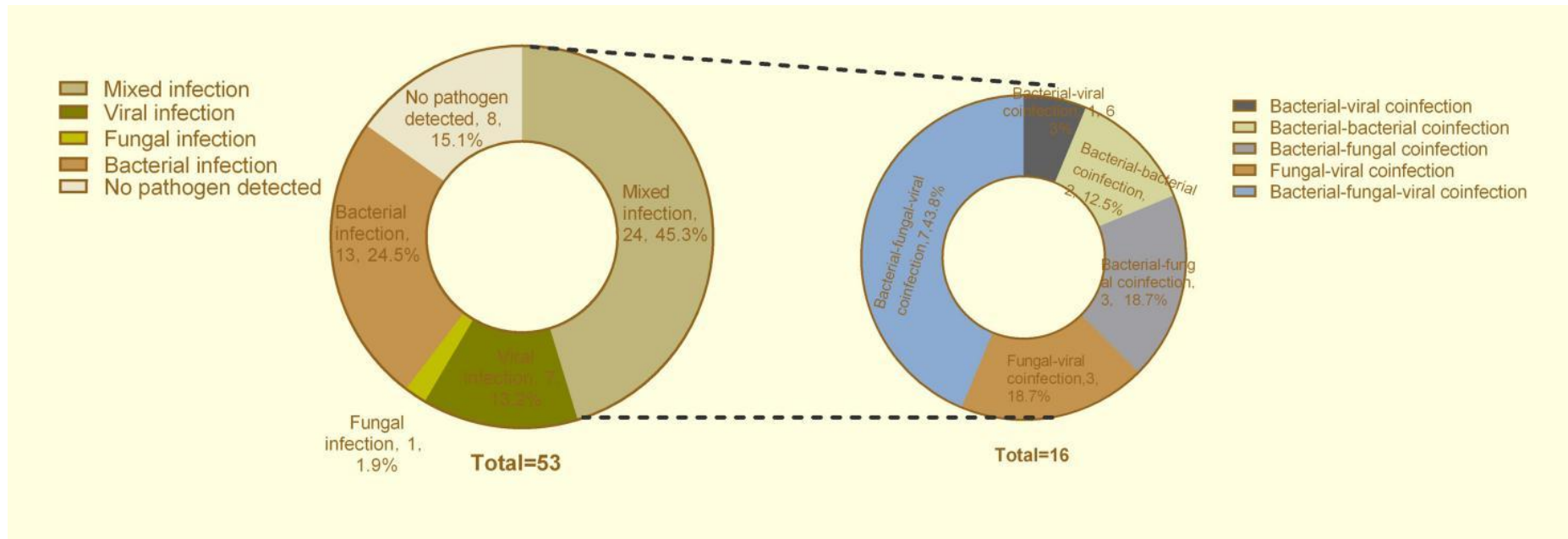

Figure S2.

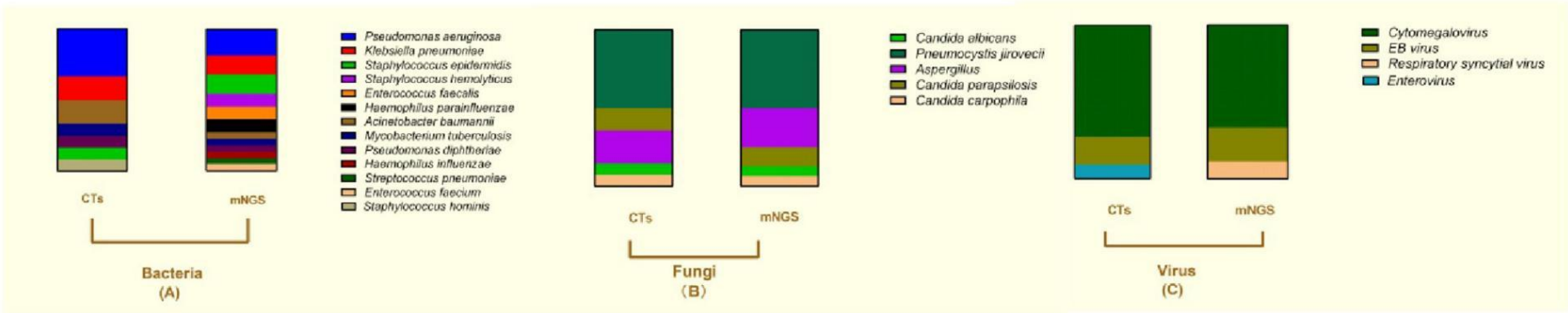

Supplement: Supplementary Materials — Figure S1. Percentage of patients with coinfections with various pathogens in immunocompromised patients. Figure S2. The comparison of mNGS and CTs for the detection of different pathogens in immunocompromised patients. Table S1. Infection identified by CTs and mNGS. [file 5884568.f1.zip › Supplementary Figures (1).pdf]
